# NCAPD2 serves as a potential prognostic biomarker for lung adenocarcinoma and promotes cell proliferation, migration, invasion and cell cycle *in vitro*

**DOI:** 10.32604/or.2024.047490

**Published:** 2024-08-23

**Authors:** PEILING WU, LIFANG ZHAO, HONGYAN ZHANG, YUEYAN LOU, DONGFANG CHEN, SHAN XUE, XUEQING LIU, HANDONG JIANG

**Affiliations:** Department of Respiratory and Critical Care Medicine, Renji Hospital, Shanghai Jiao Tong University School of Medicine, Shanghai, 200127, China

**Keywords:** NCAPD2, LUAD, Prognosis, Immune infiltration, Cell cycle

## Abstract

**Objectives:**

The pro-oncogenic effects of NCAPD2 have been extensively studied across various tumor types; however, its precise role within the context of lung adenocarcinoma (LUAD) remains elusive. This study aims to elucidate the biological functions of NCAPD2 in LUAD and unravel the underlying mechanistic pathways.

**Methods:**

Utilizing bioinformatics methodologies, we explored the differential expression of NCAPD2 between normal and tumor samples, along with its correlations with clinical-pathological characteristics, survival prognosis, and immune infiltration.

**Results:**

In the TCGA-LUAD dataset, tumor samples demonstrated significantly elevated levels of NCAPD2 expression compared to normal samples (*p* < 0.001). Clinically, higher NCAPD2 expression was notably associated with advanced T, N, and M stages, pathologic stage, gender, smoking status, and diminished overall survival (OS). Moreover, differentially expressed genes (DEGs) associated with NCAPD2 were predominantly enriched in pathways related to cell division. Immune infiltration analysis revealed that NCAPD2 expression levels were linked to the infiltration of memory B cells, naïve CD4+ T cells, activated memory CD4+ T cells, and M1 macrophages. *In vitro* experiments demonstrated that silencing NCAPD2 suppressed LUAD cell proliferation, migration, invasion, epithelial-mesenchymal transition (EMT), and cell cycle progression.

**Conclusions:**

In summary, NCAPD2 may represent a promising prognostic biomarker and novel therapeutic target for LUAD.

## Introduction

Lung cancer is a highly prevalent malignancy and remains a primary contributor to cancer-related mortality, accounting for approximately 2.2 million cases annually and causing an estimated 1.79 million fatalities [1–3]. Histologically, lung cancer is typically categorized as non-small cell lung cancer (NSCLC) or small cell lung cancer (SCLC). LUAD is the most commonly encountered subtype of NSCLC [4–6]. In comparison to other histological types, LUAD exhibits a greater tumor mutation burden (TMB) [7]. While targeted therapy and immunotherapy have shown benefits for certain LUAD patients, the overall cure and survival rates remain unsatisfactory due to the late detection and development of drug resistance [5,8]. Hence, it is imperative to augment the early diagnostic efficacy and identify novel therapeutic targets of LUAD.

NCAPD2, a non-SMC subunit of condensin I, is located at the chromosome 12p13 locus [9]. Its primary function involves segregation and alignment of chromosomes [10–12]. Condensin I and condensin II are distinct forms of condensin protein complexes found in various eukaryotic cells [13], assembling and segregating chromosomes during both mitosis and meiosis [14]. Furthermore, they may assume specific functions in the context of innate immune responses [15–17]. The regulatory influence of condensin I on gene expression has been demonstrated, and evidence suggests a potential correlation between the dysregulation of condensin I and the development of cancer [17–19]. As a component of condensin I, NCAPD2 is implicated in a range of diseases, including Alzheimer’s disease, microcephaly, Parkinson’s disease, and various neurodevelopmental disorders [20–22]. Furthermore, multiple studies have substantiated the engagement of NCAPD2 in the processes of tumorigenesis and progression [23–26]. For example, NCAPD2 is overexpressed in breast cancer and promotes its development through transcriptional activation of CDK1. Additionally, NCAPD2 has been shown to hinder autophagy, thereby facilitating the progression of colorectal cancer [27,28].

By employing bioinformatics methodologies, this investigation explored the nexus between NCAPD2 expression and clinical outcomes in LUAD patients. Our findings indicate a robust correlation between elevated NCAPD2 expression and an unfavorable prognosis in LUAD patients as well as a connection between the expression of NCAPD2 and immune infiltration. Furthermore, NCAPD2 is implicated in cell proliferation, migration, and invasion. Knockdown of NCAPD2 results in cell cycle arrest and a reduction in both c-Myc mRNA and protein expression. Consequently, the results of the study propose that NCAPD2 holds promise as a regulatory target in LUAD.

## Materials and Methods

### Data acquisition

The clinical data and gene expression profile within the TCGA-LUAD dataset were retrieved from the UCSC Xena database to investigate the expression pattern of NCAPD2 and its potential role in LUAD. The analysis included 513 tumor samples and 59 normal samples after excluding those with incomplete clinical information. Furthermore, the GSE30219 dataset from the GEO database was obtained to assess OS. All samples were divided into two groups based on the expression level of NCAPD2, either high or low.

### Expression level of NCAPD2 in LUAD

The levels of NCAPD2 expression in various types of cancer were obtained from the TIMER database [29]. Comparative assessment of NCAPD2 expression within the TCGA-LUAD dataset followed, including the difference between normal and tumor specimens, and distinctions among various T, N, M, and pathologic stages. The plot R package was utilized to visualize these comparisons. Receiver operating characteristic (ROC) curves and area under the curve (AUC) were generated by the pROC R package to evaluate the diagnostic utility of NCAPD2. The ROC curve can assess the performance of classification models and the AUC was utilized as a metric to measure the overall performance of the model [30]. Typically, an AUC value exceeding 0.5 signifies a certain degree of classification ability in the model, with a higher value closer to 1 indicating superior performance.

### Survival analysis

For evaluating the correlation between NCAPD2 expression and OS, Kaplan-Meier survival curves were constructed utilizing the survival R package. The chi-squared test was used analyze the disparity in clinical features between the NCAPD2-high and NCAPD2-low groups. Survival outcomes were explored using univariate Cox regression analysis as well as multivariate Cox regression analysis to assess the impact of NCAPD2 expression levels [31].

### Identification of DEGs and functional enrichment analysis

The analysis of NCAPD2-related DEGs was conducted using the gene expression profile from the TCGA-LUAD dataset. This profile was derived from RNA-seq data obtained from frozen primary untreated tumors collected from patients with LUAD. The identification of DEGs associated with NCAPD2 was performed using the limma R package, with significance thresholds set at adjusted *p* < 0.05 and an absolute log fold change (logFC) ≥ 1. Volcano plots and heatmaps were generated to represent the DEGs. We subsequently applied the clusterProfiler R package to conduct Gene Ontology (GO) and Kyoto Encyclopedia of Genes and Genomes (KEGG) enrichment analyses for NCAPD2-related DEGs. Terms with adjusted *p* values below 0.05 were considered statistically significant, and presented using the ggplot2 package [32]. To perform a more comprehensive investigation of the potential biological function of NCAPD2, we employe the clusterProfiler R package for gene set enrichment analysis (GSEA). This analysis utilized the c2.cp.reactome.v7.4.symbols.gmt dataset obtained from the Molecular Signatures Database [33].

### Immune infiltration analysis

To investigate the impact of NCAPD2 expression on immune infiltration in LUAD, 22 distinct types of infiltrating immune cells were scored for each sample according to the CIBERSORT algorithm [34] and the infiltration levels of immune cells were compared between the NCAPD2-high and NCAPD2-low groups. Moreover, the Spearman correlation test was employed to assess the correlation between the expression of NCAPD2 and the level of immune cell infiltration. To further substantiate the link between NCAPD2 expression and immune infiltration in LUAD, the TIMER database was explored.

### Cell culture

A549 and H1299 cell lines of LUAD were acquired from the American Type Culture Collection (ATCC) (Manassas, VA, USA) and cultured in RPMI 1640 medium (Life Technology, CA, USA) supplemented with 10% fetal bovine serum (FBS) (Gibco, Grand Island, USA) as per the provided guidelines [35].

### Small interfering RNA (siRNA) transfection

To suppress NCAPD2 expression, siRNA transfection was performed. Following the manufacturer’s guidelines, we seeded 2 × 10^5^ cells into separate wells of a 6-well plate. After 24 h, siRNA molecules specifically targeting NCAPD2 were introduced into the cells using Lipofectamine™2000 transfection reagent (Invitrogen, MA, USA). All siRNAs, including three distinct siRNAs (si-1, si-2, and si-3) targeting NCAPD2, and a siRNA negative control (NC) were procured from ObiO Technology (Shanghai, China). There are the siRNA sequences below:

NCAPD2 si-1 sense: 5′-GTAUGUUGUGCAAGAGGUACU-3′

NCAPD2 si-1 antisense: 5′-AGUACCUCUUGCACAACAUAC-3′

NCAPD2 si-2 sense: 5′-CAAAGAAGAUACUCUGCAAUU-3′

NCAPD2 si-2 antisense: 5′-AAUUGCAGAGUAUCUUCUUUG-3′

NCAPD2 si-3 sense: 5′-GGCAGACAAGUCAGUGCUAGU-3′

NCAPD2 si-3 antisense: 5′-ACUAGCACUGACUUGUCUGC-3′.

### Quantitative real-time polymerase chain reaction (RT-qPCR)

Total RNA was extracted from both untransfected and transfected cells using EZ-press RNA Purification Kit (EZBioscience, Roseville, USA). Then, HiScript III RT SuperMix for qPCR (Vazyme, Nanjing, China) was used to convert the RNA into cDNA. After that, we measured NCAPD2 expression levels using qRT-PCR, with β-actin serving as the internal reference. The formula RQ = 2^−ΔΔCt^ was used to analyze data from three independent experiments. The primer sequences are provided below:

NCAPD2-forward: 5′-TGGAGGGGTGAATCAGTATGT -3′;

NCAPD2-reverse: 5′-GCGGGATACCACTTTTATCAGG-3′

β-actin-forward: 5′-CGGGAAATCGTGCGTGAC-3′

β-actin- reverse: 5′-CAGGAAGGAAGGCTGGAAG-3′

### Western blot

Total protein was extracted from the cells using RIPA lysis buffer (Beyotime, Shanghai, China). After the protein concentrations were quantified with the Enhanced BCA Protein Assay Kit (Beyotime, Shanghai, China), equal quantities of protein were subsequently separated via SDS-PAGE, transferred to a nitrocellulose (NC) membrane and blocked at room temperature with 5% BSA. The membranes were then incubated with primary antibodies overnight at 4°C. Antibodies against NCAPD2 and β-actin were sourced from Abcam (Cambridge, UK); antibodies for vimentin, E-cadherin, CDK2, CDK4, CDK6, CyclinD1 and CyclinA2 were obtained from Cell Signaling Technology (Beverly, MA, USA) and c-Myc antibody was procured from ABclonal (Wuhan, China). Following the washing of the membrane with TBST, the application of suitable secondary antibodies for a 1-h incubation period was carried out, followed by another round of TBST washing prior to detection. All the experiments were independently conducted three times.

### CCK8 assay

Untransfected A549 and H1299 cells, as well as transfected cells, were seeded in a 96-well plate at a density of 3000 cells per well. A 10 µl volume of CCK8 reagent (Beyotime, Shanghai, China) was introduced to each well after 0, 24, 48, and 72 h, after which the cells were incubated for 2.5 h at 37°C in the absence of light. Cell viability was evaluated by measuring the optical density (OD) at a wavelength of 450 nm.

### Colony formation assay

Cells from the siNCAPD2 group and the NC group were evenly distributed into a 6-well plate respectively with a seeding density of 1000 cells per well. Subsequently, the cells were placed in a CO_2_ incubator at 37°C for 10 days, after which the medium was changed as required. Following the incubation period, the supernatant was aspirated, and the cells were fixed with a 4% paraformaldehyde solution (Beyotime, Shanghai, China) for 30 min. Afterwards, the cells were dyed with a 0.1% crystal violet solution (Beyotime, Shanghai, China), and cell quantification was conducted using ImageJ software (NIH, USA).

### Cell migration and invasion assay

For the cell migration assay, the upper compartment of the transwell chambers was initially filled with 200 μL of serum-free medium containing 2 × 10^4^ cells. The cells included untransfected A549 and H1299 cells, as well as transfected cells. The lower compartment was supplemented with 600 μl of medium containing 10% FBS. After a 48-h incubation period, the lower surface of the upper chamber was fixed with a 4% paraformaldehyde solution for 30 min, and then stained with a 0.1% crystal violet solution. Eventually, any cells that had not migrated from the upper compartment were scratched thoroughly. The results were counted by microscope. For the invasion assay, first, a diluted Matrigel solution (1:19) (BD Biosciences, NJ, USA) was introduced into the upper chamber, and the chambers were preincubated at 37°C for 4 h. The same protocol used for the migration assay was then followed.

### Flow cytometry

To conduct the cell cycle analysis, the cells were initially subjected to trypsin treatment, washed with phosphate-buffered saline (PBS), and subsequently fixed with 70% ethanol at a temperature of 4°C for 12 h. The staining procedure adhered to the protocol outlined in the cell cycle and apoptosis detection kit (Beyotime, Shanghai, China). Detection and analysis were carried out using the flow cytometer (BD Bioscience, San Jose, CA).

### Statistical analysis

SPSS 22.0 (IBM, USA) and GraphPad Prism 9.0 (La Jolla, CA, USA) were used for statistical analysis. *t*-test or Wilcoxon test was applied for group comparisons, and the Log-rank test assessed OS differences. Spearman correlation analysis investigated gene relationships, and a *p* value below 0.05 indicated statistical significance.

## Result

### NCAPD2 is highly expressed in LUAD

**Figure 1:**
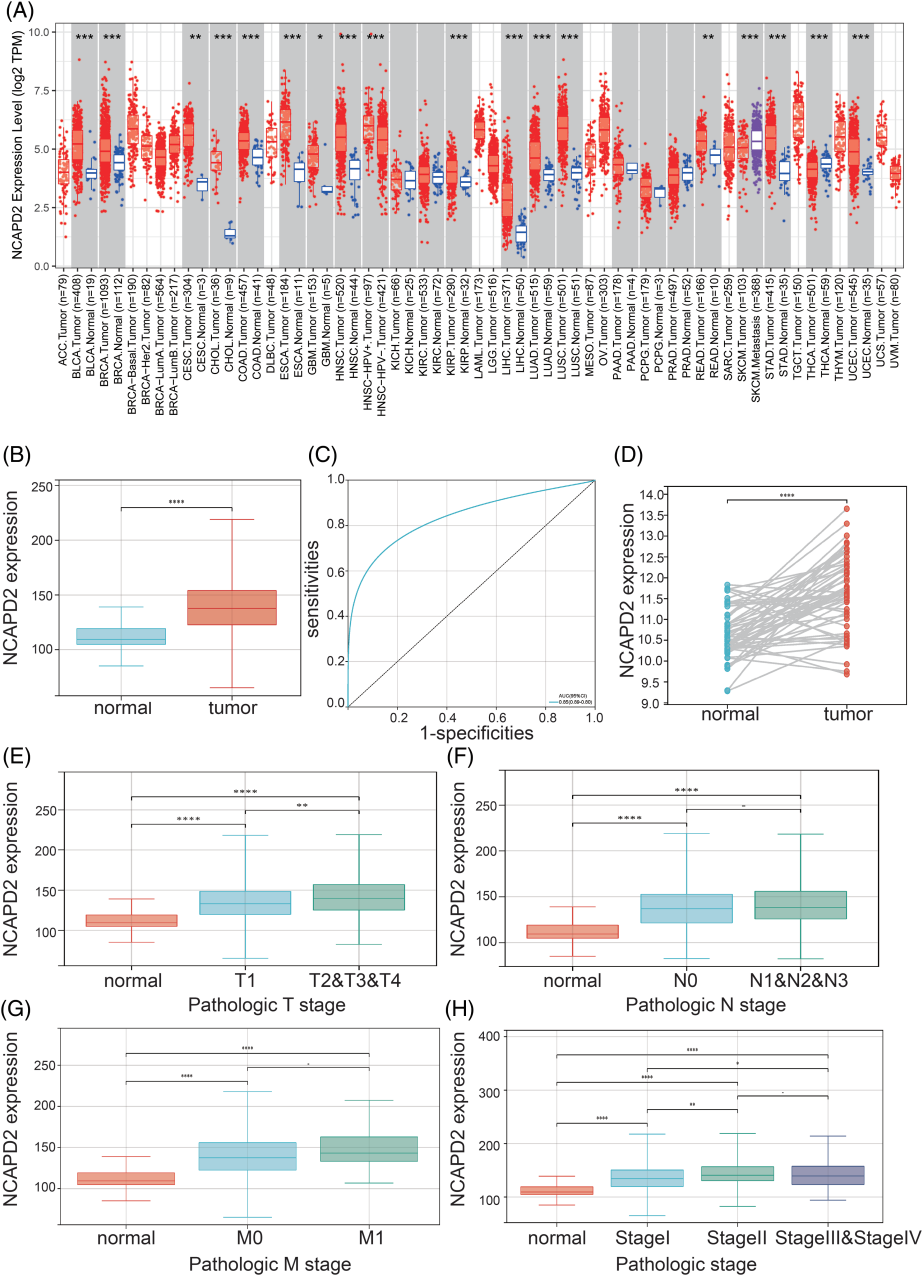
NCAPD2 expression in LUAD. (A) Pan-cancer analysis of NCAPD2 expression. (B) Comparison of NCAPD2 expression levels between normal and tumor tissues. (C) ROC curve of NCAPD2 in LUAD. (D) NCAPD2 expression levels in LUAD tissues and paired normal tissues. (E–H) NCAPD2 expression levels in different T, N and M stages and pathologic stage (*p < 0.05, **p < 0.01, ***p < 0.001; ****p < 0.0001).

Initially, TIMER 2.0 was used to assess the expression of NCAPD2 in diverse malignant tumors. In comparison to that in corresponding normal tissues, NCAPD2 demonstrated significantly elevated expression in a range of tumors, encompassing cervical squamous cell carcinoma, breast cancer, bladder cancer, bile duct cancer, head and neck squamous cell carcinoma, esophageal cancer, colon cancer, glioblastoma, lung squamous cell carcinoma, hepatocellular carcinoma, renal papillary cell carcinoma, lung adenocarcinoma, rectal adenocarcinoma, gastric cancer, thyroid cancer, and endometrial cancer (Fig. 1A). In contrast to that in normal tissues, the TCGA-LUAD dataset revealed a notable increase in NCAPD2 expression in tumor tissues (*p* < 0.001) (Fig. 1B). Moreover, to evaluate the diagnostic utility of NCAPD2 for discriminating between LUAD tissues and normal lung tissue, a ROC curve was generated, yielding an AUC of 0.85 (Fig. 1C). Additionally, compared to those in adjacent normal tissues in paired samples from TCGA-LUAD dataset, the expression levels of NCAPD2 in LUAD tissues were elevated (*p* < 0.01) (Fig. 1D). Furthermore, a significant increase in NCAPD2 expression was observed in the T2&T3&T4 stage group compared with T1 stage group. (*p* < 0.001), and both the stage II group and the stage III & stage IV group exhibited higher levels of NCAPD2 expression than did the stage I group (*p* < 0.05) (Figs. 1E–1H). These findings provide evidence of an association between elevated NCAPD2 expression and advanced T stage as well as pathologic stage in LUAD patients.

### Elevated NCAPD2 expression predicts an adverse prognosis in LUAD

**Figure 2:**
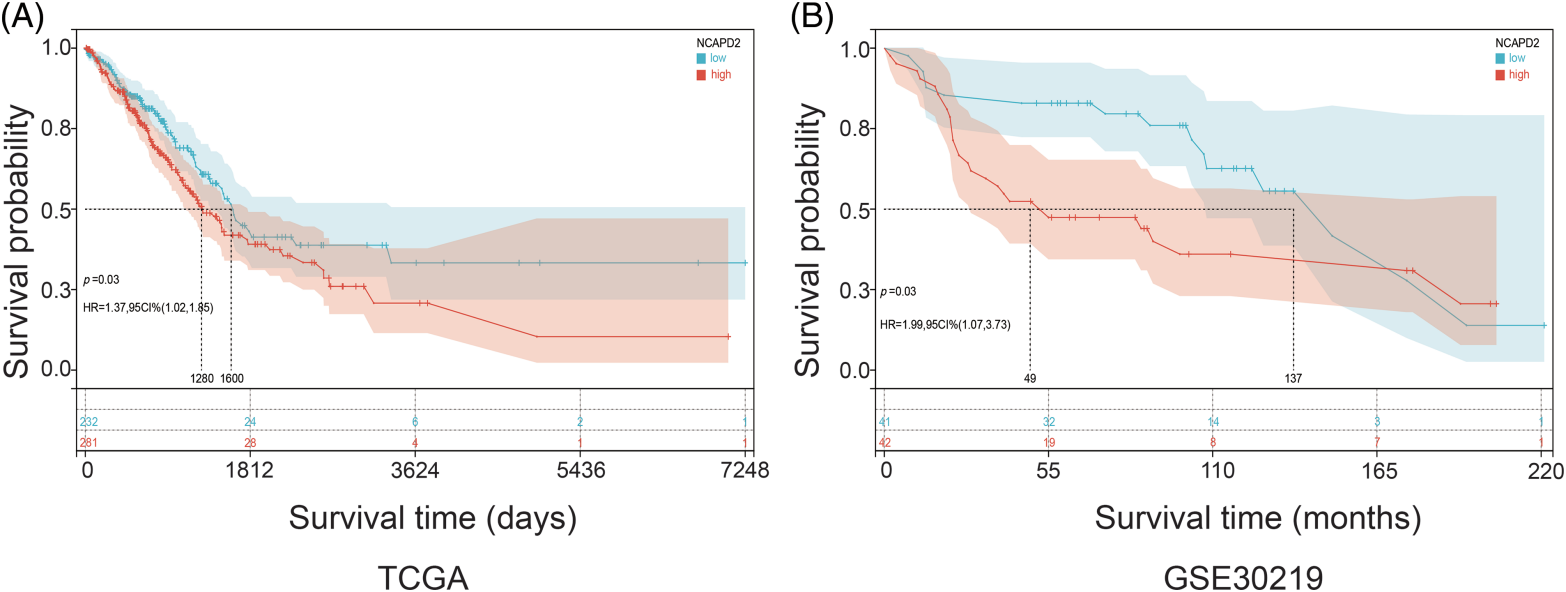
Correlation between the expression level of NCAPD2 and prognosis. (A) Kaplan–Meier survival curves: analysis of OS (days) between the NCAPD2-high group (red line) and the NCAPD2-low group (blue line) based on the TCGA-LUAD dataset. (B) Kaplan–Meier survival curves: OS (months) in 83 specimens grouped according to the median NCAPD2 expression in the GSE30219 dataset.

We gathered the expression profiles and clinical data of 513 cases in the TCGA-LUAD dataset and 83 cases in the GSE30219 dataset to determine whether NCAPD2 expression is associated with prognosis in LUAD. Analysis via the Kaplan-Meier method showed that increased NCAPD2 expression was linked to worse OS in both the TCGA-LUAD dataset (median OS: NCAPD2-high *vs*. NCAPD2-low = 1280 days *vs*. 1600 days, HR = 1.37, 95% CI 1.02–1.85, *p* < 0.05) and the GSE30219 dataset (median OS: NCAPD2-high *vs*. NCAPD2-low = 49 months *vs*. 137 months, HR = 1.99, 95% CI 1.07–3.73, *p* < 0.05) (Figs. 2A and 2B). Furthermore, strong associations were observed between high NCAPD2 expression and gender (*p* < 0.01), pathologic stage (*p* < 0.01), and smoking years (*p* < 0.01) (Table 1). The findings of the univariate Cox regression analysis demonstrated significant associations between advanced M stage (*p* = 0.005, HR = 2.15), N stage (*p* < 0.001, HR = 2.58), T stage (*p* = 0.003, HR = 1.69), pathologic stage (*p* < 0.001, HR = 2.94), and high NCAPD2 expression level (*p* = 0.035, HR = 1.37) and poorer prognosis. However, only N stage (*p* = 0.041, HR = 1.85) and T stage (*p* = 0.013, HR = 1.77) remained independent prognostic factors for LUAD according to multivariate Cox regression analysis (Table 2).

**Table 1:** Differences in clinical features between the NCAPD2-high and NCAPD2-high groups in the TCGA-LUAD cohort

Characteristics	High expression of NCAPD2 (N = 281)	Low expression of NCAPD2 (N = 232)	*p* value
Age			0.59
<=65	139 (27.63%)	108 (21.47%)	
>65	137 (27.24%)	119 (23.66%)	
Gender			1.70E-03
Female	133 (25.93%)	143 (27.88%)	
Male	148 (28.85%)	89 (17.35%)	
M stage			0.18
M0	187 (51.09%)	155 (42.35%)	
M1	17 (4.64%)	7 (1.91%)	
N stage			0.4
N0	177 (35.40%)	158 (31.60%)	
N1	59 (11.80%)	35 (7.00%)	
N2	38 (7.60%)	31 (6.20%)	
N3	1 (0.20%)	1 (0.20%)	
T stage			0.1
T1	80 (15.69%)	91 (17.84%)	
T2	162 (31.76%)	113 (22.16%)	
T3	26 (5.10%)	20 (3.92%)	
T4	10 (1.96%)	8 (1.57%)	
Pathologic stage			9.30E-03
Stage I	138 (27.33%)	142 (28.12%)	
Stage II	78 (15.45%)	42 (8.32%)	
Stage III	43 (8.51%)	37 (7.33%)	
Stage IV	18 (3.56%)	7 (1.39%)	
Number_pack_years_smoked			7.30E-03
<40	89 (25.28%)	86 (24.43%)	
>=40	116 (32.95%)	61 (17.33%)	
Tobacco_smoking_history			0.25
No	35 (7.01%)	37 (7.41%)	
Yes	242 (48.50%)	185 (37.07%)	

**Table 2:** Univariate and multivariate Cox regression analyses of clinical characteristics associated with overall survival (OS) in LUAD

		Univariate analysis			Multivariate analysis	
Characteristics	N	*p* value	HR (95% CI)	Characteristics	*p* value	HR (95% CI)
Age						
Age	513	0.352	1.01 (0.99–1.02)			
Gender						
Female	276					
Male	237	0.769	1.04 (0.78–1.39)			
M stage				M stage		
M0	342			M0		
M1	24	0.005	2.15 (1.26–3.68)	M1	0.172	1.55 (0.83–2.92)
N stage				N stage		
N0	336			N0		
N1&N2&N3	166	<0.001	2.58 (1.93–3.47)	N1&N2&N3	0.041	1.85 (1.02–3.33)
T stage				T stage		
T1	171			T1		
T2&T3&T4	339	0.003	1.69 (1.20–2.38)	T2&T3&T4	0.013	1.77 (1.13–2.80)
Pathologic stage				Pathologic stage		
Stage I	280			Stage I		
Stage II&Stage III&Stage IV	225	<0.001	2.94 (2.17–3.98)	Stage II&Stage III&Stage IV	0.397	1.32 (0.70–2.49)
NCAPD2				NCAPD2		
Low	232			Low		
High	281	0.035	1.37 (1.02–1.85)	High	0.606	1.10 (0.77–1.57)

### Functional enrichment analysis of NCAPD2-related DEGs

**Figure 3:**
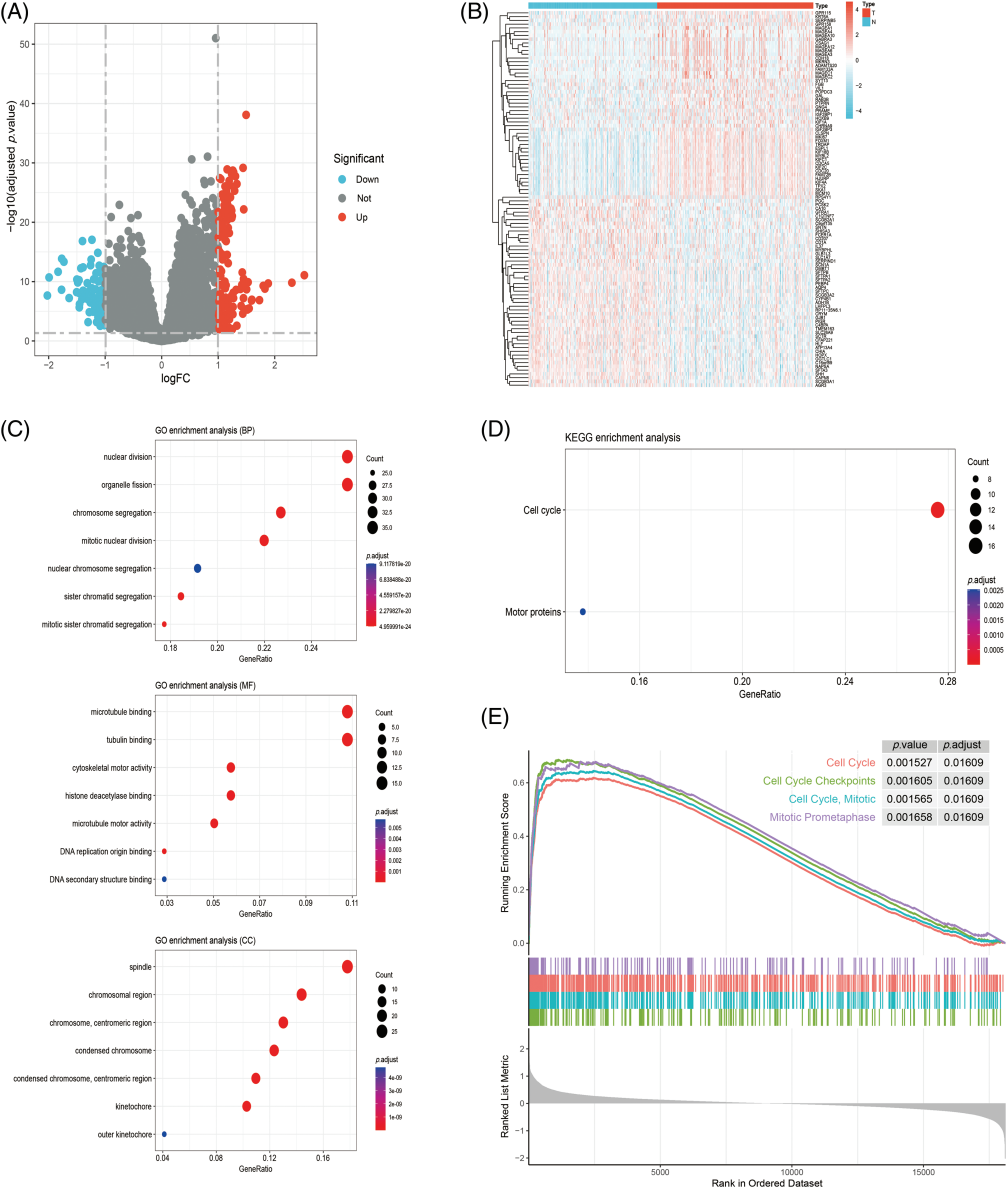
Analysis of the NCAPD2-related DEGs and their functional enrichment. (A, B) Volcano plots and heatmap of DEGs. (C, D) GO and KEGG analyses of DEGs. (E) GSEA pathway enrichment of DEGs in the Reactome database.

The analysis of DEGs identified 247 NCAPD2-related DEGs, consisting of 156 upregulated genes and 91 downregulated genes (Figs. 3A and 3B). To elucidate the biological function of the NCAPD2-related DEGs, we conducted GO function and KEGG pathway enrichment analyses, and the outcomes of the GO function enrichment analysis can be divided into three groups: biological processes (BP), cellular components (CC), and molecular functions (MF). Within the BP category, the NCAPD2-relted DEGs were significantly enriched in nuclear division and organelle fission. In CC, these genes were primarily associated with microtubule binding and tubulin binding. For MF, the enriched terms were predominantly linked to spindle and chromosomal regions (Fig. 3C). The KEGG pathway enrichment analysis primarily showed enrichment in pathways such as cell cycle (Fig. 3D). Furthermore, GSEA was employed to further explore the signaling pathways related to NCAPD2, revealing enrichment of pathways such as cell cycle, cell cycle mitotic, cell cycle checkpoints, and mitotic prometaphase (Fig. 3E).

### NCAPD2 expression is correlated with immune infiltration

**Figure 4:**
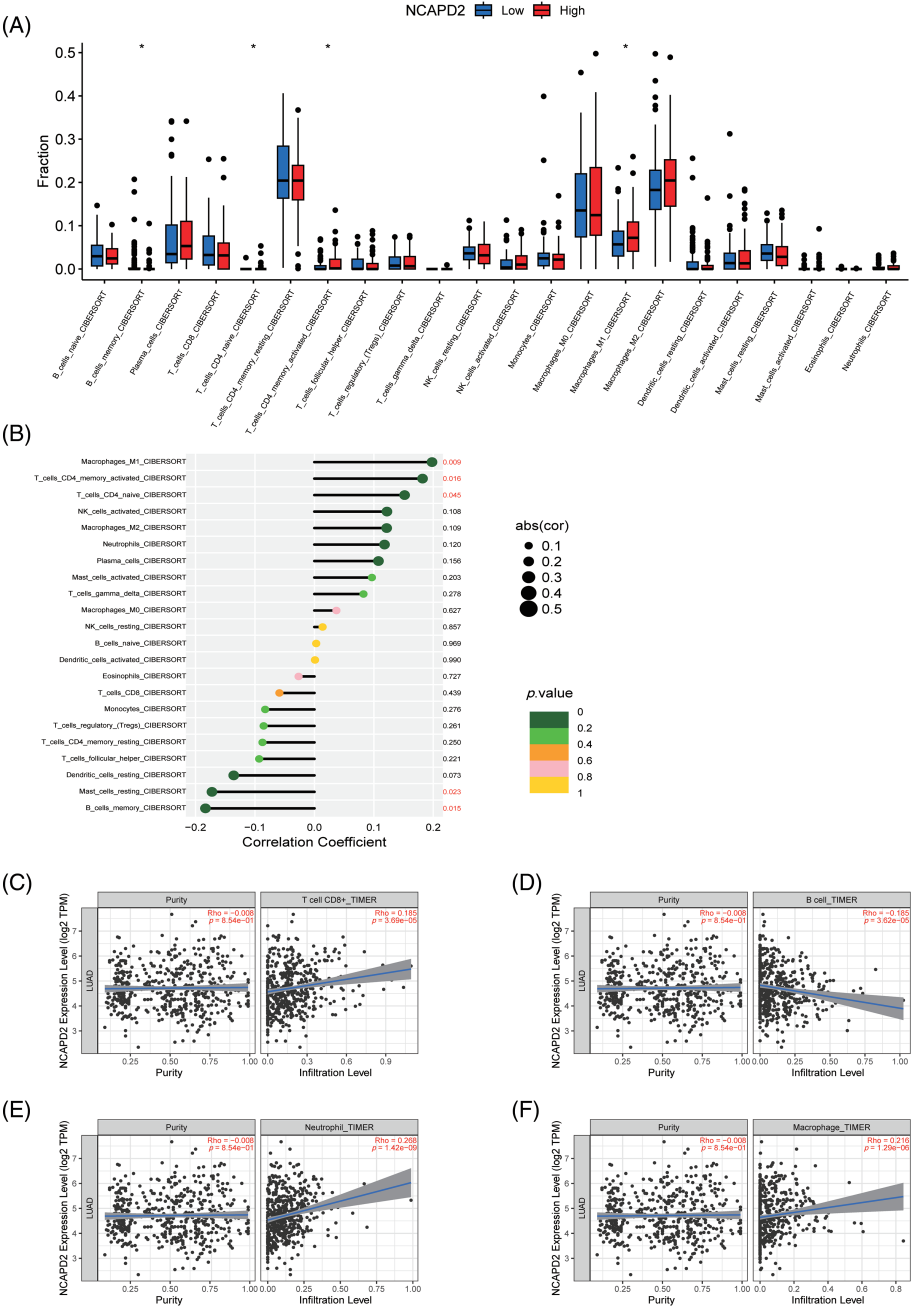
Relationship between NCAPD2 expression and immune cell infiltration. (A) Differences in immune cell abundance between the NCAPD2-high and NCAPD2-low groups. (B) Correlations between NCAPD2 expression levels and the relative abundances of 22 immune cell types. (C–F) Correlations between NCAPD2 expression and the infiltration levels of CD8+ T cells, B cells, neutrophils and macrophages in LUAD based on TIMER database. *p < 0.05.

To investigate the role of NCAPD2 in tumor immunity, the CIBERSORT algorithm was utilized to analyze the relationship between NCAPD2 expression and immune cell infiltration. The findings revealed significant differences in the infiltration levels of M1 macrophages, activated memory CD4+ T cells, naïve CD4+ T cells and memory B cells between the NCAPD2-high and NCAPD2-low groups. Furthermore, the infiltration levels of M1 macrophages, naïve CD4+ T cells and activated memory CD4+ T cells exhibited positive correlation with the NCAPD2 expression, while the infiltration level of memory B cells showed a negative correlation with NCAPD2 expression (Figs. 4A and 4B). Moreover, according to the TIMER database, the infiltration levels of CD8+ T cells (r = 0.185, *p* < 0.001), neutrophils (r = 0.268, *p* < 0.001), and macrophages (r = 0.216, *p* < 0.001) were positively correlated with NCAPD2 expression, whereas the infiltration level of B cells (r = −0.185, *p* < 0.001) was negative correlated with NCAPD2 expression (Figs. 4C–4F).

### NCAPD2 participates in cell proliferation, migration, invasion and epithelial-mesenchymal transition (EMT) in lung adenocarcinoma cells

**Figure 5:**
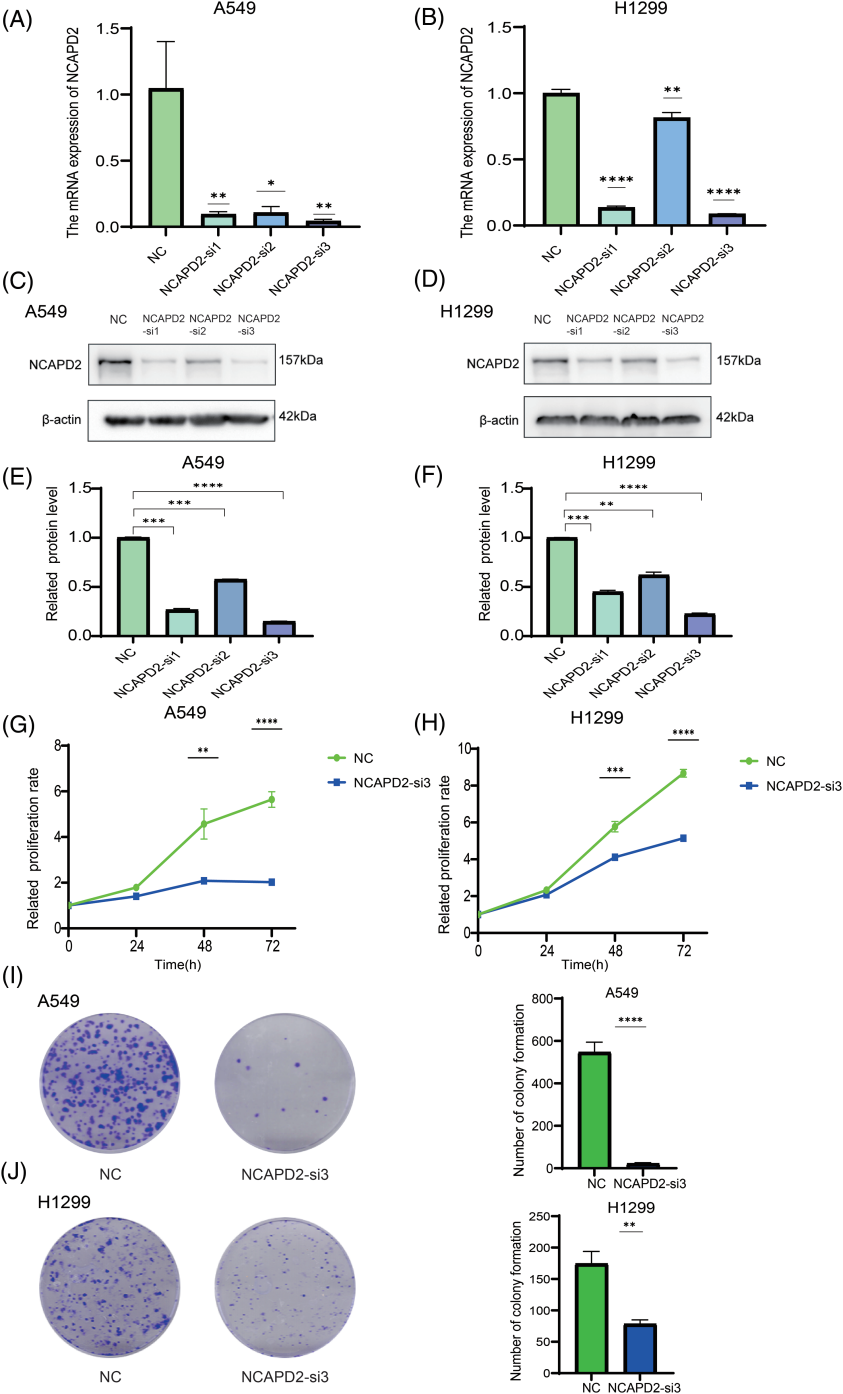
Knockdown of NCAPD2 regulates the proliferation of LUAD cells. (A, B) The mRNA expression level of NCAPD2 in A549 and HA1299 cells transfected with three different siRNAs. (C–F) The protein level of NCAPD2 in A549 and HA1299 cells transfected with three different siRNAs. (G, H) CCK8 assay for cell proliferation. (I, J) Colony formation assay for cell proliferation (*p < 0.05, **p < 0.01, ***p < 0.001, ****p < 0.0001).

**Figure 6:**
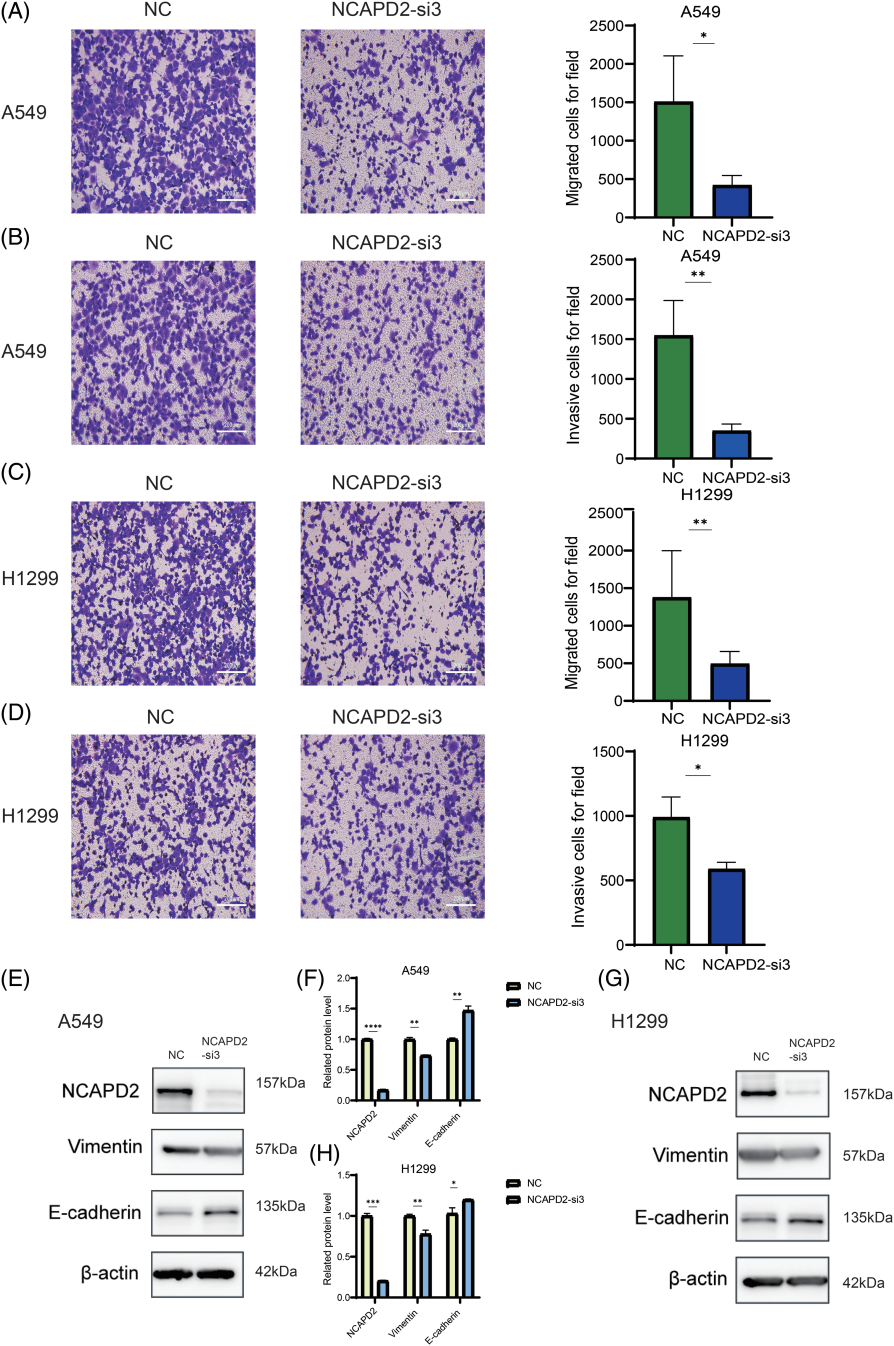
Knockdown of NCAPD2 influences LUAD cell migration, invasion and epithelial-mesenchymal transformation. (A, C) Migration assay (scale bar 200 μm). (B, D) Invasion assay (scale bar 200 μm). (E–H) The levels of EMT-related proteins were measured via western blotting (*p < 0.05, **p < 0.01, ***p < 0.001,****p < 0.0001).

Our previous research illustrated a correlation between elevated NCAPD2 expression and an unfavorable prognosis, thus, the function of NCAPD2 was tested in LUAD cells *in vitro*. To assess the efficiency of NCAPD2 knockdown, the mRNA and protein expression levels of NCAPD2 in cells were measured after siRNA transfection. The results indicated that si-3 had the highest knockdown efficiency (Figs. 5A–5F), consequently, si-3 was selected for subsequent experiments. Assays for CCK8 and colony formation were performed to evaluate cell proliferation capacity. Overall, the proliferation of A549 and H1299 cells was strongly inhibited by the knockdown of NCAPD2, in contrast to that in the NC group (Figs. 5G–5J). The migration assay primarily evaluates the locomotive ability of cells within a two-dimensional plane, while the invasion assay specifically explores the invasive potential of cells by assessing their capacity to penetrate a supportive matrix that mimics the intricate extracellular environment encountered *in vivo*. The migratory and invasive capabilities of A549 and H1299 cells were significantly suppressed following the knockdown of NCAPD2 (Figs. 6A–6D). The effect of NCAPD2 on EMT was further evaluated in the context of tumor metastasis. Compared with the NC group, the siNCAPD2 group exhibited higher levels of the E-cadherin protein and lower levels of the vimentin protein both in A549 cells (Figs. 6E, 6F) and H1299 cells (Figs. 6G, 6H), indicating a shift toward an EMT-inhibiting phenotype.

### Knockdown of NCAPD2 inhibits the cell cycle and decreases the c-Myc expression level in LUAD cells

**Figure 7:**
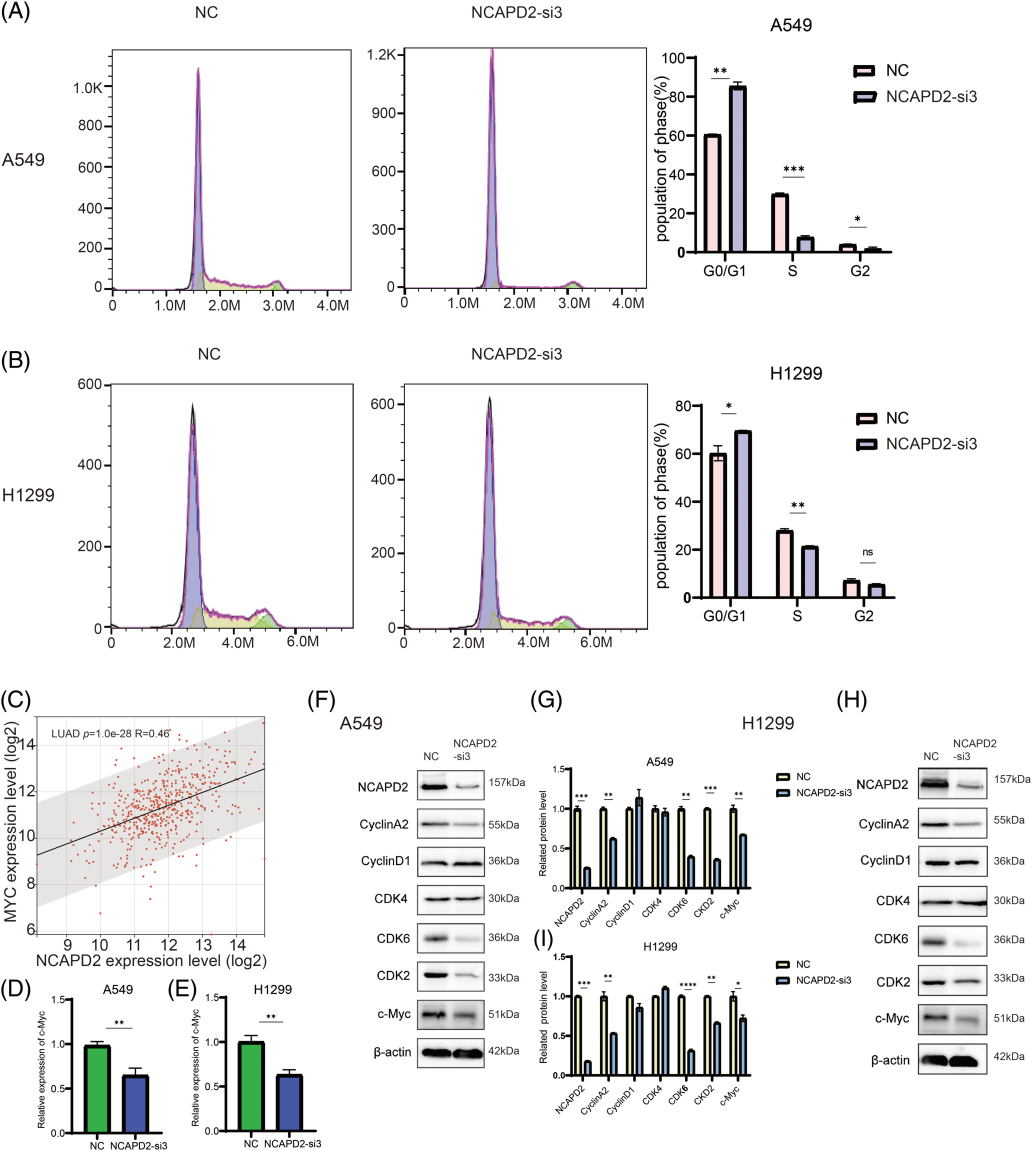
Knockdown of NCAPD2 regulates the cell cycle. (A, B) The cell cycle distribution was examined by flow cytometry. (C) Correlation between the expression level of NCAPD2 and the expression level of c-Myc. (D, E) The expression levels of c-Myc were measured via RT-qPCR. (F–I) The levels of cell cycle-related proteins were measured via western blotting (*p < 0.05, **p < 0.01, ***p < 0.001, ****p < 0.0001).

The influence of NCAPD2 on the cell cycle progression of LUAD cells was investigated using flow cytometry. Compared to that in the NC group, knocking down NCAPD2 increased the proportion of A549 and H1299 cells in the G0/G1 phase and reduced the proportion of cells in the S phase. The percentage of A549 cells in the G2/M phase decreased slightly, while the percentage of H1299 cells in the G2/M phase did not significantly differ (Figs. 7A and 7B). The Western blot results indicated that NCAPD2 knockdown led to decreased protein levels of CyclinA2, CDK2 and CDK6 in both cell types, but there was no significant difference in CyclinD1 or CDK4 levels. Previous research has indicated that NCAPD2 co-expressed genes are enriched in the MYC target pathway in various tumors [23]. Additionally, c-Myc and its induced genes play crucial roles in cell cycle control and cell growth [36]. Thus, we analyzed the correlation between NCAPD2 expression and c-Myc expression in TCGA-LUAD cohort. Our analysis revealed a positive correlation between the expression levels of NCAPD2 and c-Myc (R = 0.47, *p* < 0.001) (Fig. 7C). Moreover, following NCAPD2 knockdown, both the mRNA and protein levels of c-Myc exhibited the most significant decreases (Figs. 7D–7I). Based on these results, knockdown of NCAPD2 induces cell cycle arrest in the G0/G1 and S phases and may do so by regulating c-Myc expression levels.

## Discussion

Condensin, a multisubunit protein complex within the structural maintenance of chromosomes (SMC) complex family, plays a pivotal role in orchestrating chromosome structure regulation. It involves diverse processes, including DNA repair and recombination, gene regulation, and chromosome segregation [14]. NCAPD2 assumes the role of a non-SMC subunit in condensin I, influencing the function of another non-SMC subunit of condensin I, sister chromatid separation, and chromosome alignment during metaphase [12]. Although previous studies have hinted at a potential connection between NCAPD2 and the prognosis of LUAD, its precise function and underlying molecular mechanisms remain elusive [23]. The objective of this research was to clarify the involvement of NCAPD2 in the progression of LUAD, with the intention of identifying a new potential prognostic biomarker and treatment targe.

Initially, we compared the differential expression of NCAPD2 between normal and LUAD tissues, confirming the upregulated expression of NCAPD2 in LUAD, and its increased expression related to advanced T, N and M stages and pathologic stage. Kaplan–Meier and univariate Cox regression analyses consistently indicated that elevated NCAPD2 expression serves as a predictive factor for worse overall survival (OS). The results of enrichment analyses in GO and KEGG indicated that NCAPD2 was associated with cell cycle processes and the GSEA results reinforced the findings. Subsequent *in vitro* experiments provided further validation by demonstrating that NCAPD2 knockdown induced cell cycle arrest in the G1/G0 and S phases, corroborating the outcomes of the G0 and KEGG analyses. Western blot results indicated a decrease in cyclin A, CDK2, and CDK6 protein levels upon NCAPD2 knockdown. Previous research has suggested that dysregulation of the transition from the G1 phase of cell cycle to S phase promotes oncogenesis [37]. CyclinA is associated with CDK2 and impacts the S phase of the cell cycle and overexpression of cyclin A expedites the transition of G1 cells into the S phase in mammalian cells [38,39]. These findings imply that the involvement of NCAPD2 in regulating the cell cycle may contribute to the development of LUAD. Furthermore, our investigation revealed a decrease in c-Myc mRNA and protein levels following NCAPD2 knockdown *in vitro*. Some studies have suggested that aberrant overexpression of MYC is common in NSCLC [40]. The c-Myc oncogene family, encoding nuclear phosphoproteins, has vital functions in cell proliferation, loss of differentiation, apoptosis, tumorigenesis, cancer cell reprogramming and chemoresistance [41–43]. Moreover, c-Myc is believed to exert dual effects by both stimulating and inhibiting specific components of the cell cycle machinery and is correlated with two distinct genetic pathways that control cell progression through the G1 phase [42]. These findings indicate that NCAPD2 may regulate the cell cycle through c-Myc. However, the specific underlying mechanisms need to be further researched.

Currently, there is a growing acknowledgment of the pivotal role played by the immune system in the progression of tumors [44]. Multiple studies have shown that NCAPD2 impacts immune cell infiltration in various tumors, nevertheless, its precise role in LUAD remains uncertain [23]. Therefore, our study delves into the intricate relationship between NCAPD2 and immune cell infiltration, utilizing the CIBERSORT algorithm and validating our findings through the TIMER database. The results obtained from the CIBERSORT algorithm were consistently aligned with those from the TIMER database. Specifically, B cell infiltration in LUAD was negatively correlated with NCAPD2 expression, while macrophage infiltration was positively correlated with NCAPD2 expression. Several studies emphasized the pivotal role of tumor-associated macrophages (TAMs) as key components in the tumor microenvironment of NSCLC. TAMs not only exert immunosuppressive effects that promote immune escape but also facilitate tumor cell proliferation, invasion, and migration [45]. However, tumor-associated B cells possess the capacity to differentiate into plasma cells within lung and generate antibodies specifically target the tumor, thereby identifying and combating tumor-related antigens. Moreover, the presence of follicular B cells and plasma cells has been associated with improved long-term survival outcomes in lung cancer, suggesting the protective function of antibodies and plasma cells in antitumor immunity [46]. Overall, in LUAD, high expression of NCAPD2 appears to play an immunosuppressive role.

*In vitro*, knocking down NCAPD2 can inhibit the proliferation, migration, invasion, and EMT of LUAD cells. The EMT serves as the foundation for the metastasis of epithelial malignancies. By augmenting cellular vitality and invasiveness, EMT enhances the migratory potential of tumor cells. Furthermore, EMT contributes to immunosuppression in LUAD, through a reduction in T-cell infiltration and promotion of T-cell exhaustion. This establishes EMT as a pivotal mechanism for immune resistance in cancers [47–49].

## Conclusions

In conclusion, this study illustrated the role of NCAPD2 in LUAD prognosis and provided experimental evidence that NCAPD2 promotes tumor development. Prior studies have indicated the overexpression of NCAPD2 in a range of tumors beyond just LUAD. In-depth investigations into the mechanisms of NCAPD2 promoting cancer have been conducted in breast cancer and colorectal cancer [27,28]. The findings from these studies underscore a potential general pro-oncogenic effect of NCAPD2, transcending the confines of specific cancer types. Given the widespread implication of NCAPD2 in various cancers, it emerges as a promising therapeutic target in the realm of cancer treatment. Future endeavors could involve the investigation of small molecule inhibitors designed to selectively target NCAPD2. Furthermore, exploring the potential synergistic effects of combining NCAPD2 targeting with immunotherapy holds considerable promise for advancing cancer therapy.

## Data Availability

The authors affirm that the data backing the discovers of this study can be found within the article.

## References

[1] Thai, A. A., Solomon, B. J., Sequist, L. V., Gainor, J. F., Heist, R. S. (2021). Lung cancer. Lancet*,* 398 *(*10299*),* 535–554. 10.1016/S0140-6736(21)00312-3; 34273294

[2] Chen, P., Liu, Y., Wen, Y., Zhou, C. (2022). Non-small cell lung cancer in China. Cancer Communications*,* 42 *(*10*),* 937–970. 10.1002/cac2.v42.10. 36075878 PMC9558689

[3] Freeman, B., Mamallapalli, J., Bian, T., Ballas, K., Lynch, A. et al. (2023). Opportunities and challenges of kava in lung cancer prevention. International Journal of Molecular Sciences*,* 24 *(*11*),* 9539. 10.3390/ijms24119539; 37298489 PMC10253622

[4] Barta, J. A., Powell, C. A., Wisnivesky, J. P. (2019). Global epidemiology of lung cancer. Annals of Global Health*,* 85 *(*1*),* 8. 10.5334/aogh.2419; 30741509 PMC6724220

[5] Herbst, R. S., Morgensztern, D., Boshoff, C. (2018). The biology and management of non-small cell lung cancer. Nature*,* 553 *(*7689*),* 446–454. 10.1038/nature25183; 29364287

[6] Zheng, M. (2016). Classification and pathology of lung cancer. Surgical Oncology Clinics of North America*,* 25 *(*3*),* 447–468. 10.1016/j.soc.2016.02.003; 27261908

[7] Cancer Genome Atlas Research Network (2014). Comprehensive molecular profiling of lung adenocarcinoma. Nature*,* 511 *(*7511*),* 543–550. 10.1038/nature13385; 25079552 PMC4231481

[8] Miller, M., Hanna, N. (2021). Advances in systemic therapy for non-small cell lung cancer. BMJ (Clinical research ed.)*,* 375*,* 2363. 10.1136/bmj.n2363 34753715

[9] Li, Y., Chu, L. W., Li, Z., Yik, P. Y., Song, Y. Q. (2009). A study on the association of the chromosome 12p13 locus with sporadic late-onset alzheimer’s disease in chinese. Dementia and Geriatric Cognitive Disorders*,* 27 *(*6*),* 508–512. 10.1159/000218740; 19451718

[10] Martin, C. A., Murray, J. E., Carroll, P., Leitch, A., Mackenzie, K. J. et al. (2016). Mutations in genes encoding condensin complex proteins cause microcephaly through decatenation failure at mitosis. Genes & Development*,* 30 *(*19*),* 2158–2172. 10.1101/gad.286351.116; 27737959 PMC5088565

[11] Ball, A. R., Schmiesing, J. A., Zhou, C., Gregson, H. C., Okada, Y. et al. (2023). Identification of a chromosome-targeting domain in the human condensin subunit CNAP1/hCAP-D2/Eg7. Molecular and Cellular Biology*,* 22 *(*16*),* 5769–5781. 10.1128/MCB.22.16.5769-5781.2002 PMC133980 12138188

[12] Watrin, E., Legagneux, V. (2023). Contribution of hCAP-D2, a non-SMC subunit of condensin I, to chromosome and chromosomal protein dynamics during mitosis. Molecular and Cellular Biology*,* 25 *(*2*),* 740–750. 10.1128/MCB.25.2.740-750.2005 PMC543417 15632074

[13] Hirano, T. (2012). Condensins: Universal organizers of chromosomes with diverse functions. Genes & Development*,* 26 *(*15*),* 1659–1678. 10.1101/gad.194746.112; 22855829 PMC3418584

[14] Paul, M. R., Hochwagen, A., Ercan, S. (2018). Condensin action and compaction. Current Genetics*,* 65 *(*2*),* 407–415; 30361853 10.1007/s00294-018-0899-4 PMC6421088

[15] Wang, Q., Wang, C., Li, N., Liu, X., Ren, W. et al. (2018). Condensin Smc4 promotes inflammatory innate immune response by epigenetically enhancing NEMO transcription. Journal of Autoimmunity*,* 92*,* 67–76. 10.1016/j.jaut.2018.05.004; 29803706

[16] Longworth, M. S., Walker, J. A., Anderssen, E., Moon, N.S., Gladden, A. et al. (2012). A shared role for RBF1 and dCAP-D3 in the regulation of transcription with consequences for innate immunity. PLoS Genetics*,* 8 *(*4*),* e1002618. 10.1371/journal.pgen.1002618; 22496667 PMC3320600

[17] Ward, J. R., Khan, A., Torres, S., Crawford, B., Nock, S. et al. (2022). Condensin I and condensin II proteins form a LINE-1 dependent super condensin complex and cooperate to repress LINE-1. Nucleic Acids Research*,* 50 *(*18*),* 10680–10694. 10.1093/nar/gkac802; 36169232 PMC9561375

[18] Kagami, Y., Yoshida, K. (2016). The functional role for condensin in the regulation of chromosomal organization during the cell cycle. Cellular and Molecular Life Sciences*,* 73 *(*24*),* 4591–4598. 10.1007/s00018-016-2305-z; 27402120 PMC11108269

[19] Zhang, T., Paulson, J. R., Bakhrebah, M., Kim, J. H., Nowell, C. et al. (2016). Condensin I and II behaviour in interphase nuclei and cells undergoing premature chromosome condensation. Chromosome Research*,* 24 *(*2*),* 243–269. 10.1007/s10577-016-9519-7; 27008552

[20] Lee, J. H., Cheng, R., Rogaeva, E., Meng, Y., Stern, Y. et al. (2008). Further examination of the candidate genes in chromosome 12p13 locus for late-onset Alzheimer disease. Neurogenetics*,* 9 *(*2*),* 127–138. 10.1007/s10048-008-0122-8; 18340469 PMC2635895

[21] Lin, Y., Zeng, C., Lu, Z., Lin, R., Liu, L. (2019). A novel homozygous splice-site variant of NCAPD2 gene identified in two siblings with primary microcephaly: The second case report. Clinical Genetics*,* 96 *(*1*),* 98–101. 10.1111/cge.2019.96.issue-1. 31056748

[22] Zhang, P., Liu, L., Huang, J., Shao, L., Wang, H. et al. (2014). Non-SMC condensin I complex, subunit D2 gene polymorphisms are associated with Parkinson’s disease: A Han Chinese study. Genome*,* 57 *(*5*),* 253–257. 10.1139/gen-2014-0032; 25166511

[23] Dong, X., Liu, T., Li, Z., Zhai, Y. (2023). Non-SMC condensin I complex subunit D2 (NCAPD2) reveals its prognostic and immunologic features in human cancers. Aging*,* 15 *(*14*),* 7237–7257. 10.18632/aging.v15i14. 37498296 PMC10415567

[24] Guan, Y. J., Ma, J. Y., Song, W. (2019). Identification of circRNA-miRNA–mRNA regulatory network in gastric cancer by analysis of microarray data. Cancer Cell International*,* 19 *(*1*),* 1–9. 31346318 10.1186/s12935-019-0905-z PMC6636116

[25] Zhao, Q., Zhang, Y., Shao, S., Sun, Y., Lin, Z. (2021). Identification of hub genes and biological pathways in hepatocellular carcinoma by integrated bioinformatics analysis. PeerJ*,* 9*,* e10594. 10.7717/peerj.10594; 33552715 PMC7821758

[26] Solár, P., Sytkowski, A. J. (2011). Differentially expressed genes associated with cisplatin resistance in human ovarian adenocarcinoma cell line A2780. Cancer Letters*,* 309 *(*1*),* 11–18. 10.1016/j.canlet.2011.05.008; 21676537

[27] He, J., Gao, R., Yang, J., Li, F., Fu, Y. et al. (2023). NCAPD2 promotes breast cancer progression through E2F1 transcriptional regulation of CDK1. Cancer Science*,* 114 *(*3*),* 896–907. 10.1111/cas.v114.3. 35348268 PMC9986070

[28] Jing, Z., He, X., Jia, Z., Sa, Y., Yang, B. et al. (2021). NCAPD2 inhibits autophagy by regulating Ca^2+^/CAMKK2/AMPK/mTORC1 pathway and PARP-1/SIRT1 axis to promote colorectal cancer. Cancer Letters*,* 520*,* 26–37. 10.1016/j.canlet.2021.06.029; 34229059

[29] Li, T., Fu, J., Zeng, Z., Cohen, D., Li, J. et al. (2020). TIMER2.0 for analysis of tumor-infiltrating immune cells. Nucleic Acids Research*,* 48 *(*W1*),* W509–W514. 10.1093/nar/gkaa407; 32442275 PMC7319575

[30] Wang, S., Li, D., Petrick, N., Sahiner, B., Linguraru, M. G. et al. (2015). Optimizing area under the ROC curve using semi-supervised learning. Pattern Recognition*,* 48 *(*1*),* 276–287. 10.1016/j.patcog.2014.07.025; 25395692 PMC4226543

[31] Shi, Y., Wang, J., Huang, G., Zhu, J., Jian, H. et al. (2022). A novel epithelial-mesenchymal transition gene signature for the immune status and prognosis of hepatocellular carcinoma. Hepatology International*,* 16 *(*4*),* 906–917. 10.1007/s12072-022-10354-3; 35699863 PMC9349121

[32] Yu, G., Wang, L. G., Han, Y., He, Q. Y. (2012). clusterProfiler: An R package for comparing biological themes among gene clusters. Omics: A Journal of Integrative Biology*,* 16 *(*5*),* 284–287. 10.1089/omi.2011.0118; 22455463 PMC3339379

[33] Liberzon, A., Subramanian, A., Pinchback, R., Thorvaldsdottir, H., Tamayo, P. et al. (2011). Molecular signatures database (MSigDB) 3.0. Bioinformatics*,* 27 *(*12*),* 1739–1740. 10.1093/bioinformatics/btr260; 21546393 PMC3106198

[34] Newman, A. M., Liu, C. L., Green, M. R., Gentles, A. J., Feng, W. et al. (2015). Robust enumeration of cell subsets from tissue expression profiles. Nature Methods*,* 12 *(*5*),* 453–457. 10.1038/nmeth.3337; 25822800 PMC4739640

[35] Baust, J. M., Buehring, G. C., Campbell, L., Elmore, E., Harbell, J. W. et al. (2017). Best practices in cell culture: An overview. Vitro Cellular & Developmental Biology—Animal*,* 53 *(*8*),* 669–672. 10.1007/s11626-017-0177-7; 28808859

[36] Baluapuri, A., Wolf, E., Eilers, M. (2020). Target gene-independent functions of MYC oncoproteins. Nature Reviews Molecular Cell Biology*,* 21 *(*5*),* 255–267. 10.1038/s41580-020-0215-2; 32071436 PMC7611238

[37] Bertoli, C., Skotheim, J. M., de Bruin, R. A. M. (2013). Control of cell cycle transcription during G1 and S phases. Nature Reviews Molecular Cell Biology*,* 14 *(*8*),* 518–528. 10.1038/nrm3629; 23877564 PMC4569015

[38] Rosenberg, A. R., Zindy, F., Le Deist, F., Mouly, H., Métézeau, P. et al. (1995). Overexpression of human cyclin a advances entry into S phase. Oncogene*,* 10 *(*8*),* 1501–1509; 7731704

[39] Yam, C. H., Fung, T. K., Poon, R. Y. (2002). Cyclin A in cell cycle control and cancer. Cellular and Molecular Life Sciences*,* 59 *(*8*),* 1317–1326. 10.1007/s00018-002-8510-y; 12363035 PMC11337442

[40] Massó-Vallés, D., Beaulieu, M. E., Soucek, L. (2020). MYC, MYCL, and MYCN as therapeutic targets in lung cancer. Expert Opinion on Therapeutic Targets*,* 24 *(*2*),* 101–114. 10.1080/14728222.2020.1723548; 32003251

[41] Fatma, H., Maurya, S. K., Siddique, H. R. (2022). Epigenetic modifications of c-MYC: Role in cancer cell reprogramming, progression and chemoresistance. Seminars in Cancer Biology*,* 83*,* 166–176. 10.1016/j.semcancer.2020.11.008; 33220458

[42] Nasi, S., Ciarapica, R., Jucker, R., Rosati, J., Soucek, L. (2001). Making decisions through Myc. FEBS Letters*,* 490 *(*3*),* 153–162. 10.1016/S0014-5793(01)02118-4; 11223030

[43] Pelengaris, S., Khan, M., Evan, G. (2002). c-MYC: More than just a matter of life and death. Nature Reviews Cancer*,* 2 *(*10*),* 764–776. 10.1038/nrc904; 12360279

[44] Olson, A. L., Gifford, A. H., Inase, N., Fernández Pérez, E. R., Suda, T. (2018). The epidemiology of idiopathic pulmonary fibrosis and interstitial lung diseases at risk of a progressive-fibrosing phenotype. European Respiratory Review*,* 27 *(*150*),* 180077. 10.1183/16000617.0077-2018; 30578336 PMC9489016

[45] Conway, E. M., Pikor, L. A., Kung, S. H. Y., Hamilton, M. J., Lam, S. et al. (2016). Macrophages, inflammation, and lung cancer. American Journal of Respiratory and Critical Care Medicine*,* 193 *(*2*),* 116–130. 10.1164/rccm.201508-1545CI; 26583808

[46] Germain, C., Gnjatic, S., Tamzalit, F., Knockaert, S., Remark, R. et al. (2014). Presence of B cells in tertiary lymphoid structures is associated with a protective immunity in patients with lung cancer. American Journal of Respiratory and Critical Care Medicine*,* 189 *(*7*),* 832–844. 10.1164/rccm.201309-1611OC; 24484236

[47] Chae, Y. K., Chang, S., Ko, T., Anker, J., Agte, S. et al. (2018). Epithelial-mesenchymal transition (EMT) signature is inversely associated with T-cell infiltration in non-small cell lung cancer (NSCLC). Scientific Reports*,* 8 *(*1*),* 2918. 10.1038/s41598-018-21061-1; 29440769 PMC5811447

[48] Xiao, G. Y., Tan, X., Rodriguez, B. L., Gibbons, D. L., Wang, S. et al. (2023). EMT activates exocytotic Rabs to coordinate invasion and immunosuppression in lung cancer. Proceedings of the National Academy of Sciences*,* 120 *(*28*),* e2220276120. 10.1073/pnas.2220276120; 37406091 PMC10334751

[49] Zhu, L., Zeng, Q., Wang, J., Deng, F., Jin, S. (2023). Cathepsin V drives lung cancer progression by shaping the immunosuppressive environment and adhesion molecules cleavage. Aging*,* 23 *(*15*),* 13961–13979. 10.18632/aging.205278 PMC10756122 38078882

